# Efficacy of ozone with steroid injection therapy in lumbar disc herniation: meta-analysis

**DOI:** 10.3389/fmed.2026.1891165

**Published:** 2026-08-17

**Authors:** Yi Zhou, Hongyu Yu

**Affiliations:** School of Nursing, Jinzhou Medical University, Liaoning, China

**Keywords:** lumbar disc herniation, meta-analysis, ozone, pain, steroid

## Abstract

**Background:**

The efficacy of ozone combined with steroid injection for lumbar disc herniation remains uncertain. This systematic review and meta-analysis aimed to investigate the efficacy of Ozone with steroid injection therapy in the treatment of lumbar disc herniation.

**Methods:**

PubMed, Embase, Web of Science, and Wanfang were searched from database inception to July 20, 2026. Comparative studies evaluating ozone combined with steroid injection in patients with lumbar disc herniation were included. Mean differences (MDs) were used for continuous outcomes, and odds ratios (ORs) were used for dichotomous outcomes, with corresponding 95% confidence intervals (CIs). Heterogeneity was assessed using Cochran's Q test and the *I*^2^ statistic.

**Results:**

Seven comparative studies involving 751 patients were included. Meta-analysis showed that Ozone combined with steroid injection was associated with a greater reduction in pain intensity than control interventions (MD = −2.27, 95% CI: −2.57 to −1.97). The combined treatment was also associated with a higher study-defined treatment success rate (OR = 3.84, 95% CI: 2.56 to 5.77). However, no statistically significant between-group difference was observed in Oswestry Disability Index (ODI) (MD = −0.51, 95% CI: −2.82 to 1.81). Safety reporting was limited, precluding quantitative synthesis of adverse events.

**Conclusions:**

Ozone combined with steroid injection may improve pain intensity and treatment success in patients with lumbar disc herniation, but its additional benefit for functional disability remains uncertain. Given the limited number of studies, mixed study designs, and heterogeneous treatment protocols, the findings should be considered preliminary and interpreted cautiously.

## Introduction

Lumbar disc herniation (LDH) is a major cause of low back pain and lumbosacral radiculopathy, resulting in pain, disability, reduced quality of life, and considerable socioeconomic burden (1). Although conservative treatments are effective in many patients, persistent radicular pain remains common, and surgery is not always appropriate for patients with mild-to-moderate symptoms or those seeking less invasive alternatives (2, 3). Consequently, image-guided minimally invasive interventions have become increasingly important in the management of symptomatic LDH.

Oxygen–ozone therapy has emerged as a potential minimally invasive treatment for LDH (4). Intradiscal ozone is thought to oxidize proteoglycans in the nucleus pulposus, reduce disc hydration and volume, and thereby decrease mechanical compression of nerve roots (5). It may also exert anti-inflammatory and analgesic effects by modulating local oxidative stress and inflammatory mediators (6). Steroid injection, in contrast, mainly targets periradicular inflammation by reducing cytokine release, nerve root edema, and inflammatory pain (7). Therefore, the combination of ozone and steroid injection may offer complementary effects by simultaneously addressing disc-related compression and nerve root inflammation.

However, the clinical benefit of this combined approach remains controversial. A randomized study reported that adding corticosteroid to oxygen–ozone injection produced greater clinical improvement at 3 weeks; however, no significant between-group differences were observed at 6 or 12 months (8). Conversely, another randomized controlled trial comparing ozone plus steroids, magnesium sulfate plus steroids, and steroids alone suggested that ozone as an adjunct to steroid injection may improve pain and disability in patients with lumbar disc prolapse-related radicular pain (9). Recent clinical evidence has further explored steroid alone vs. steroid plus ozone during epiduroscopic treatment, indicating continued interest in this combined strategy (10). Therefore, we aimed to assess the efficacy of ozone with steroid injection therapy in patients with lumbar disc herniation.

## Methods

This meta-analysis was conducted in accordance with the Preferred Reporting Items for Systematic Reviews and Meta-Analyses (PRISMA) 2020 statement (11).

### Search strategy

A comprehensive literature search was performed in PubMed, Embase, Web of Science, and Wanfang, from database inception to July 20, 2026. The search strategy combined Medical Subject Headings (MeSH) terms and keywords: lumbar disc herniation, ozone, steroid. Additional records were identified by manually screening the reference lists of included studies and relevant reviews. No language restrictions were applied. The detailed search strategies for all databases are presented in Supplementary Table 1.

### Inclusion and exclusion criteria

The inclusion and exclusion criteria were defined according to the PICOS framework. Population: Adult patients diagnosed with lumbar disc herniation based on clinical manifestations and imaging findings, including magnetic resonance imaging or computed tomography. Intervention: Ozone combined with steroid injection therapy. Comparator: Steroid injection alone. Outcomes: Studies reporting at least one clinically relevant outcome, including pain intensity, functional disability, clinical response rate, or adverse events. Pain intensity was assessed using the visual analog scale (VAS) or numerical rating scale, and functional disability was commonly assessed using the Oswestry Disability Index (ODI). Study design: Randomized controlled trials were prioritized. Controlled clinical studies were also considered eligible if the intervention, comparator, baseline characteristics, and outcome data were clearly reported. Studies were excluded if they met any of the following criteria: Case reports, review, conference abstracts; involving cervical or thoracic disc herniation without separate data for lumbar disc herniation; studies evaluating ozone therapy without steroid injection; studies with duplicated populations or overlapping datasets.

### Data extraction and risk-of-bias assessment

Two reviewers independently extracted data using a standardized data extraction form. The following information was collected from each included study: first author, publication year, country, study design, sample size, patient age, intervention details, comparator details, injection route, ozone concentration, steroid type and dose, use of local anesthetic, follow-up duration, outcome measures. The methodological quality of randomized controlled trials was assessed using the Cochrane Collaboration's Risk of Bias Assessment Tool. Non-randomized comparative studies were assessed using the ROBINS-I tool (12), which evaluates bias due to confounding, participant selection, intervention classification, deviations from intended interventions, missing data, outcome measurement, and selection of the reported result.

### Statistical analysis

Meta-analysis was performed using Review Manager version 5.2 and Stata version 12.0. When multiple follow-up assessments were reported, data from the longest available follow-up were extracted for the primary meta-analysis. The odds ratio (OR) or mean differences (MD), and the 95% confidence interval (CI) was used to estimate the interval range of the effect size. Heterogeneity was assessed using the chi-square test and the *I*^2^ statistic. An *I*^2^ value of 25%, 50%, and 75% was considered to indicate low, moderate, and high heterogeneity, respectively. A fixed-effect model was used when clinical and statistical heterogeneity were low. Otherwise, a random-effects model was used. Sensitivity analysis was performed to test the stability of the pooled results. A two-sided *P* value < 0.05 was considered statistically significant.

## Results

### Study selection and characteristics

**Figure 1:**
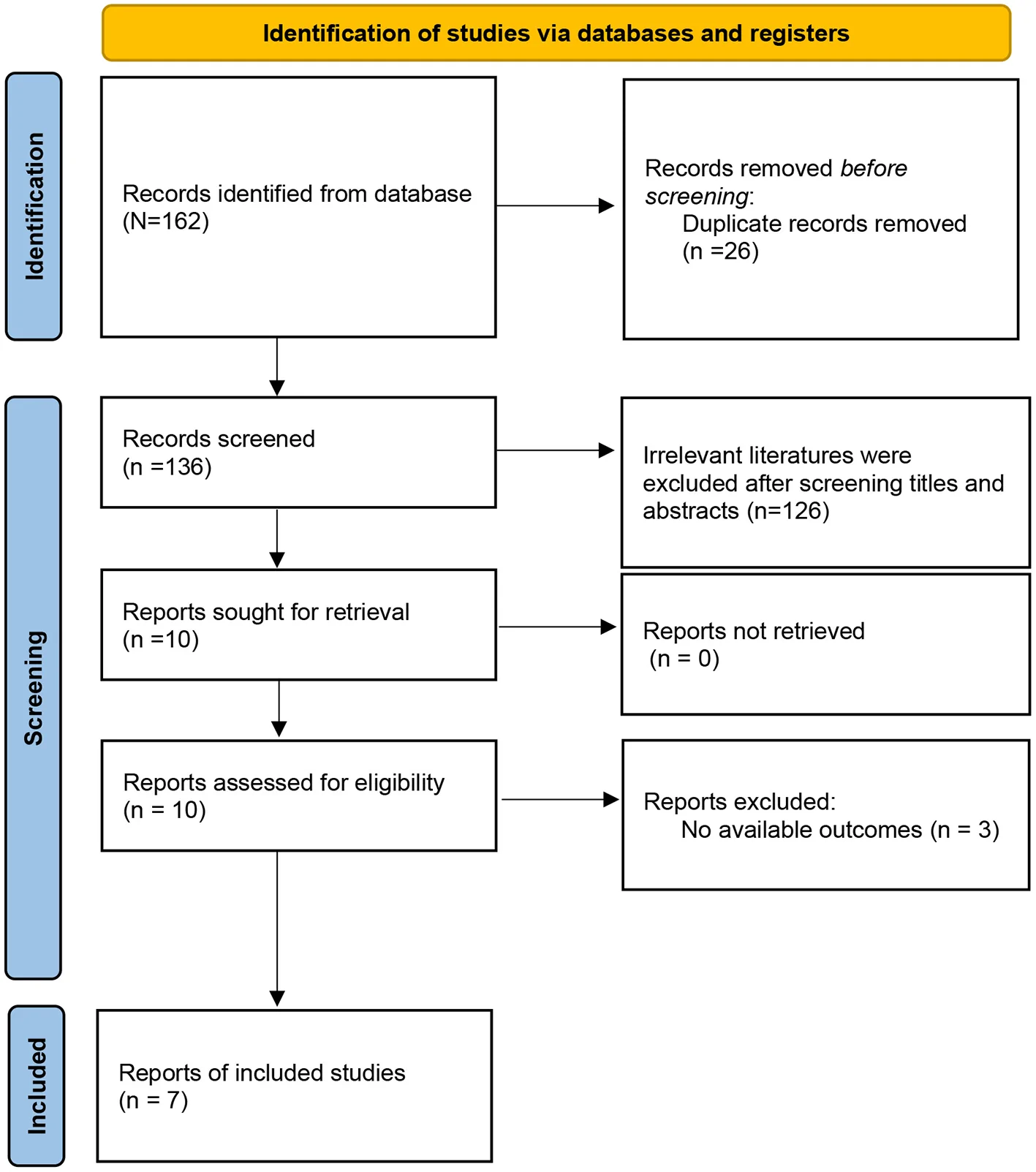
Flow diagram.

A total of 162 records were identified through database searching. After removal of duplicates, 136 records were screened by title and abstract, and 103 articles were excluded because they were clearly irrelevant. The full texts of 10 studies were assessed for eligibility. Finally, 7 studies (9, 10, 13–17) involving 751 patients were included in the meta-analysis (Figure 1). The included studies were published between 2007 and 2025 and were conducted in Italy, Türkiye, China, Korea, and Egypt. The mean age of participants ranged from approximately 40 to 67 years. Regarding interventions, the treatment group received ozone injection, while the control group mainly received steroid injection with or without local anesthetic. Follow-up duration ranged from 1 to 32.4 months. In terms of outcome assessment, pain intensity was evaluated mainly by VAS, while functional disability was assessed using the ODI or FRI. In addition, several studies reported treatment success rate as an outcome measure (Table 1). Adverse-event reporting was limited across the included studies. Several studies explicitly reported no major treatment-related complications, whereas the remaining studies did not provide detailed safety data.

**Table 1:** Baseline characteristics.

References	Country	Study type	Mean age	Sample size	Ozone treatment	Steroids treatment	Follow- up (months)	Outcomes
Bruno et al. (16)	Italy	RCT	47.52/48.49	30/30	27 μg/mL, intradiscal	Steroids (betamethasone, 4 mg) with local anesthetic	1	VAS, ODI
Bayram et al. (10)	Türkiye	CT	60.9/61.3	41/35	20 μg/mL, epidural space	Steroids (dexamethasone, 40 mg)	12	VAS, ODI
Yang et al. (17)	China	RCT	66.6/62.9	65/67	30 μg/mL, paravertebral	Steroids (triamcinolone acetonide, 0.5%) with local anesthetic	6	VAS, success rate
Gallucci et al. (13)	Italy	RCT	40/41	82/77	25 μg/mL, intraforaminal	Steroids (triamcinolone acetonide 80 mg) with local anesthetic	6	success rate
Perri et al. (15)	Italy	RCT	44.4/43.8	77/77	28 μg/mL, intraforaminal	Steroids (betamethasone, 4 mg) with local anesthetic	6	success rate
Zhang et al. (14)	China	RCT	53/58	40/40	50 μg/mL, intradiscal	Steroids (betamethasone) with local anesthetic	32.4	success rate
Fathy et al. (9)	Egypt	RCT	58.93/54.91	45/45	25 μg/mL, transforaminal epidural injection	Steroids (betamethasone 7 mg) with local anesthetic	6	NRS, ODI, FRI

RCT, randomized controlled trial; CT, controlled trial NRS, numeric rating scale; ODI, oswestry back disability, FRI, functional rating index; VAS, visual analog scale.

### Risk of bias assessment results

**Figure 2:**
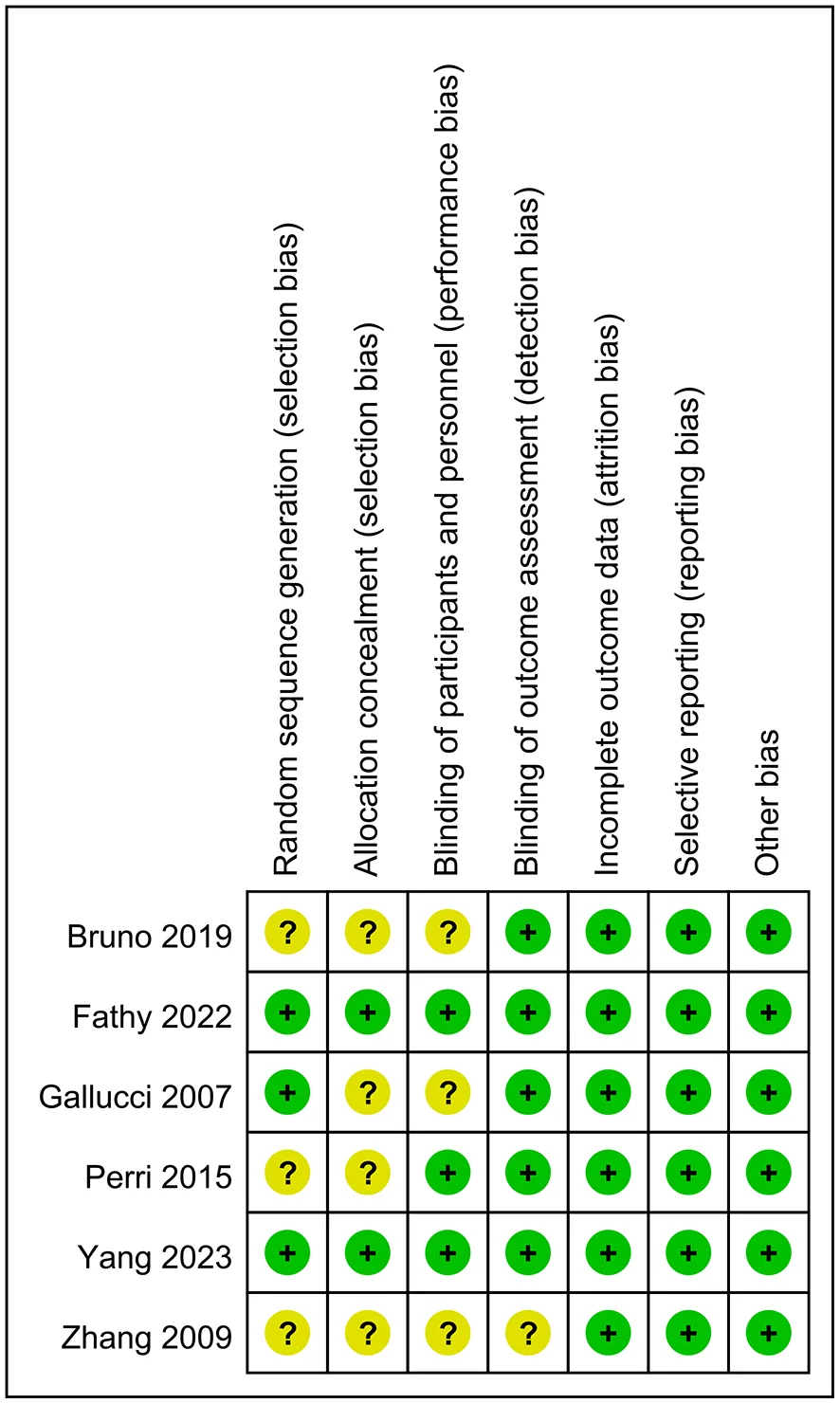
Summary of risk of bias.

Risk-of-bias judgments varied across the included studies (Figure 2). Among the randomized controlled trials, most concerns were classified as unclear risk, mainly because several studies did not provide sufficient methodological details on randomization, allocation concealment, or blinding procedures. The domains of incomplete outcome data, selective reporting, and other bias were consistently rated as low risk across all included studies. Among the randomized controlled trials, one study has a moderate risk of bias because of the confounding, participant selection, and performance bias according to the ROBINS-I tool (Supplementary Table 2).

### Effect of ozone with steroid injection on pain intensity

**Figure 3:**
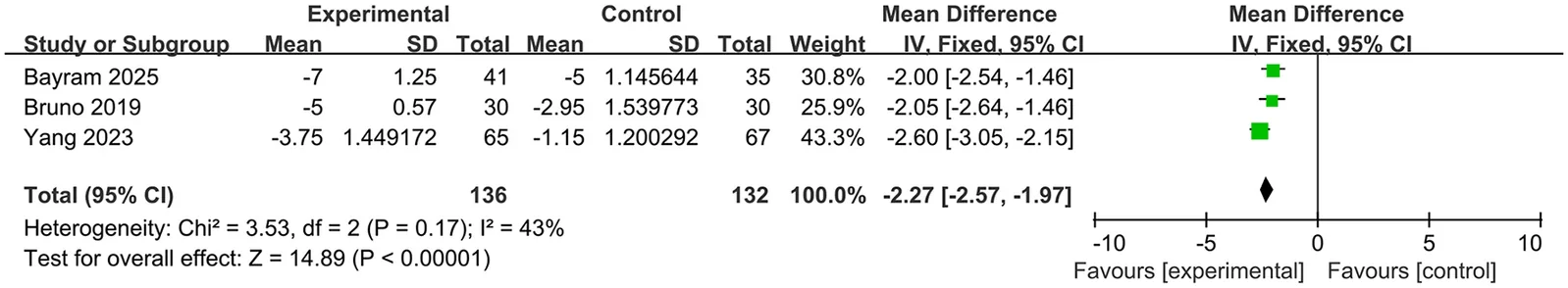
Forest plot of the meta-analysis for the VAS.

Pain intensity was assessed using VAS in 3 studies (Figure 3). Compared with the control group, ozone combined with steroid injection significantly reduced pain intensity in patients with lumbar disc herniation (MD = −2.27, 95% CI: −2.57 to −1.97, *P* < 0.001). Heterogeneity among studies was low (*I*^2^ = 43%, *P* = 0.17), and a fixed-effect model was used. However, the small number of studies and differences in treatment protocols, outcome definitions, and follow-up durations warrant cautious interpretation of the pooled estimates.

### Effect of ozone with steroid injection on functional disability

**Figure 4:**
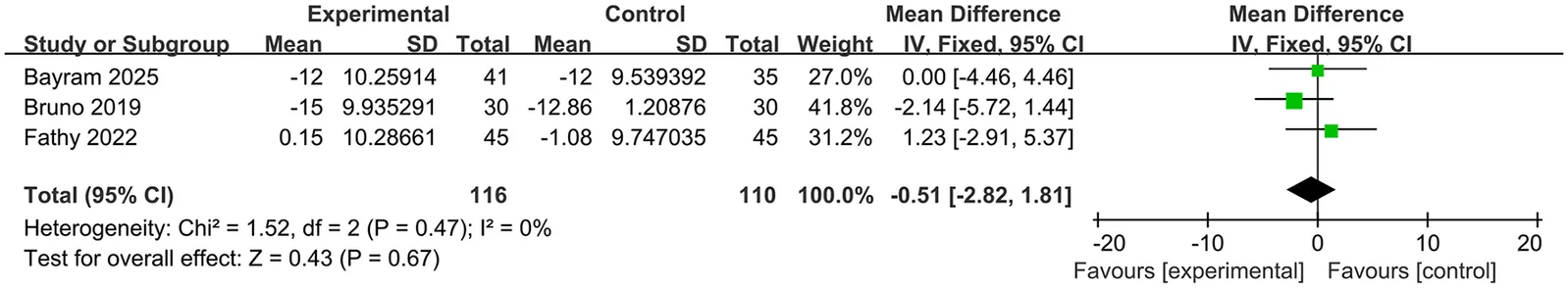
Forest plot of the meta-analysis for the ODI.

Functional disability was evaluated using the ODI in 3 studies (Figure 4). The pooled results indicated no significant difference in functional disability between ozone combined with steroid injection and the control treatment (MD = −0.51, 95% CI: −2.82 to 1.81, *P* = 0.67). As heterogeneity was low (*I*^2^ = 0%, *P* = 0.47), a fixed-effect model was used.

### Treatment success rate

**Figure 5:**
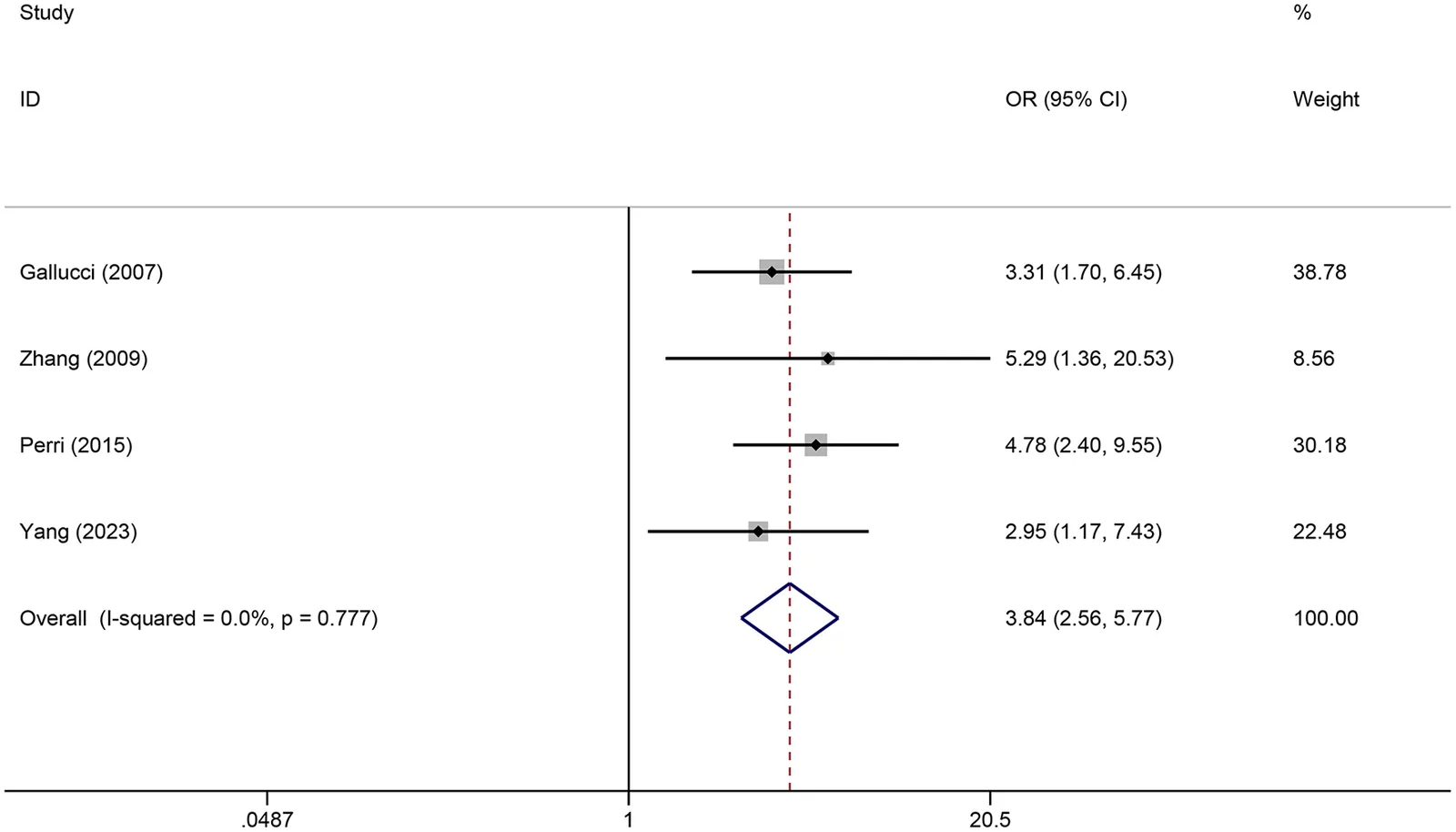
Forest plot of the meta-analysis for the treatment success rates.

The treatment success rate was reported in four studies (Figure 5). The criteria were not uniform: one study defined success as an excellent or good outcome according to the modified MacNab criteria, one used pain relief with a follow-up VAS score of ≤ 2, and two applied ODI-based criteria. The pooled analysis showed that ozone combined with steroid injection was associated with higher odds of treatment success rate than the control interventions (OR = 3.84, 95% CI: 2.56–5.77, *P* < 0.001). No statistically detectable heterogeneity was observed (*I*^2^ = 0%, *P* = 0.777).

### Sensitivity analyses

Sensitivity analyses excluding studies with a high or unclear risk of bias yielded pooled estimates that were consistent in direction and magnitude with the primary analyses. Leave-one-out analyses also showed that no individual study materially altered the pooled results.

## Discussion

The present meta-analysis suggests that ozone combined with steroid injection may be associated with greater pain relief and a higher treatment success rate than the control interventions in patients with lumbar disc herniation. However, these findings should be considered preliminary. Only a small number of studies contributed to each pooled analysis, and the included studies differed in injection route, ozone concentration, steroid regimen, and follow-up duration. Although statistical heterogeneity was not detected for pain intensity or treatment success, the small number of studies and differences in treatment protocols, comparator interventions, and follow-up durations limit confidence in the consistency and generalizability of the pooled estimates.

A plausible explanation for the superior pain relief observed in the combined treatment group is that the two components may exert complementary therapeutic effects. Steroid injection primarily acts by suppressing local inflammation and reducing nerve root edema, thereby providing short-term symptomatic improvement (4, 18). Ozone, in contrast, may contribute through additional mechanisms, such as reducing disc volume, improving the local inflammatory microenvironment, and decreasing chemical irritation around the affected nerve root (19). Therefore, the combination of these two modalities may achieve a broader therapeutic effect than control treatment alone, which could explain the significant reduction in pain intensity and the higher overall success rate observed in this analysis.

Our findings differ from some previous reports, particularly with respect to functional recovery. In our pooled analysis, ozone combined with steroid injection did not significantly improve ODI, despite producing a clear analgesic benefit. This discrepancy may reflect differences in study design, comparator selection, and outcome framework. Several earlier studies evaluated ozone-based therapy against usual conservative management or reported pre–post improvement after treatment, whereas our analysis specifically examined the added value of combining ozone with steroid injection relative to control interventions. In this context, Zhang et al. reported that adding corticosteroid to intradiscal and intraforaminal oxygen–ozone treatment produced greater early clinical improvement; however, no significant between-group differences were observed at 6- or 12-month follow-up, suggesting that the additional benefit of steroid co-administration may not persist over the longer term (14). The comparator interventions were not completely uniform across the included studies. Bayram et al. was the only study that used steroid alone as the control treatment, whereas the remaining studies administered steroid in combination with a local anesthetic (10). This difference may have influenced the observed treatment effects, particularly for short-term pain outcomes, because local anesthetics can provide rapid but transient analgesia independent of the anti-inflammatory effect of corticosteroids. In addition, a 2024 systematic review by Llombart-Blanco et al. (20) suggested that ozone therapy showed only limited benefit over usual care, with a small short-term functional advantage but inconsistent findings for pain and overall clinical outcomes. Llombart-Blanco et al. (20) evaluated ozone infiltration broadly for low back pain and included comparisons with usual care and several alternative interventions, whereas the present analysis was restricted to patients with lumbar disc herniation and focused specifically on the incremental effect of combining ozone with steroid injection. The comparator groups in our analysis primarily received steroid alone, allowing assessment of whether the combined regimen offered an additional benefit over either component.

The divergence between pain relief and functional disability in our analysis is clinically plausible. Pain intensity may respond relatively rapidly to local anti-inflammatory and decompressive effects, whereas functional disability is a broader endpoint influenced by multiple factors, including symptom duration, baseline impairment, muscle deconditioning, psychosocial status, and rehabilitation adherence (21, 22). Therefore, even when pain is significantly reduced, ODI may not improve to the same extent within the available follow-up period. This interpretation is also compatible with the broader ozone literature, in which pain reduction tends to be more consistently observed than durable functional restoration (23). Measurement characteristics may also have contributed to the non-significant ODI result. The ODI may exhibit a floor effect in patients with relatively mild baseline disability, leaving limited scope for further improvement; conversely, clustering of scores among patients with severe disability may reduce the sensitivity of the scale to detect modest functional changes (24). In addition, functional improvement may occur more slowly than analgesic effects. Asher et al. found that ODI outcomes obtained at 3 months did not consistently represent individual functional status at 12 months after lumbar spine surgery, indicating that relatively short follow-up may underestimate delayed recovery (25).

Although no treatment-related complications were reported in included studies. Ozone injection is an invasive procedure, and rare but clinically important complications have been described. A recent case report documented pneumoperitoneum following ozone therapy for low back pain, illustrating the possibility of procedure-related gas dissemination (26). Other case reports have described serious infection and nerve-root injury after spinal oxygen–ozone injection (27). Nevertheless, isolated case reports cannot establish the incidence of these events or quantify the comparative risk of ozone-based treatment. Future trials should prospectively define, systematically monitor, and report adverse events using standardized safety outcomes.

Several limitations should be acknowledged. First, the number of included studies was small, and only three studies contributed to the analyses of pain intensity and functional disability, which limits statistical power and the precision of pooled estimates. Second, the control interventions, ozone protocols, steroid regimens, and follow-up durations were not completely uniform across studies, which may have introduced clinical heterogeneity even though statistical heterogeneity was low. Third, because fewer than 10 studies were included, formal assessment of publication bias was not performed. Thus, small-study effects and selective outcome reporting cannot be excluded, and the pooled estimates may overstate the true treatment effect. Fourth, several risk-of-bias domains, particularly allocation concealment and blinding, were judged as unclear because the original studies provided insufficient methodological detail. These uncertainties reduce confidence in the pooled estimates, as inadequate allocation concealment may introduce selection bias and incomplete blinding may influence subjective outcomes such as pain intensity, disability, and treatment success. Therefore, residual methodological bias cannot be excluded, and the findings should be interpreted cautiously. Fifth, the included evidence comprised both randomized controlled trials and non-randomized comparative studies. The non-randomized studies may be more susceptible to selection bias, thereby reducing the certainty of the pooled estimates. Therefore, the present findings should be interpreted with appropriate caution, and further high-quality randomized controlled trials with standardized protocols and longer follow-up are still needed.

Overall, the current evidence suggests that ozone combined with steroid injection may improve pain intensity and treatment success in patients with lumbar disc herniation, but its benefit for functional disability remains uncertain. Further high-quality studies are required to validate these findings.

## Data Availability

The original contributions presented in the study are included in the article/Supplementary material, further inquiries can be directed to the corresponding author.
