# Supplementary material for: Efficacy of ozone with steroid injection therapy in lumbar disc herniation: meta-analysis
Source: Front Med (Lausanne). 2026 Aug 17;13:1891165. doi: 10.3389/fmed.2026.1891165 (PMC13527453; doi:10.3389/fmed.2026.1891165)
Supplement: Supplementary file 1 [file Table_1.docx]

**Supplementary Table 1. The details of the search strategies.**

| Database | Search strategy |
| --- | --- |
| PubMed | ("lumbarised"[All Fields] OR "lumbarization"[All Fields] OR "lumbarized"[All Fields] OR "lumbars"[All Fields] OR "lumbosacral region"[MeSH Terms] OR ("lumbosacral"[All Fields] AND "region"[All Fields]) OR "lumbosacral region"[All Fields] OR "lumbar"[All Fields]) AND "disc"[All Fields] AND ("hernia"[MeSH Terms] OR "hernia"[All Fields] OR "herniation"[All Fields] OR "herniations"[All Fields] OR "herniate"[All Fields] OR "herniated"[All Fields] OR "herniates"[All Fields] OR "herniating"[All Fields]) AND ("ozonated"[All Fields] OR "ozonating"[All Fields] OR "ozonation"[All Fields] OR "ozonations"[All Fields] OR "ozone"[Supplementary Concept] OR "ozone"[All Fields] OR "ozone"[MeSH Terms] OR "ozone s"[All Fields] OR "ozonization"[All Fields] OR "ozonized"[All Fields] OR "ozonizer"[All Fields]) AND ("steroidal"[All Fields] OR "steroidals"[All Fields] OR "steroidic"[All Fields] OR "steroids"[Supplementary Concept] OR "steroids"[All Fields] OR "steroid"[All Fields] OR "steroids"[MeSH Terms])  Identified 26 articles, Date: July 20. 2026. |
| Embase | ('lumbar disc herniation'/exp OR 'lumbar disc herniation' OR (lumbar AND disc AND ('herniation'/exp OR herniation))) AND ('ozone'/exp OR ozone) AND ('steroid'/exp OR steroid)  Identified 90 articles, Date: July 20. 2026. |
| Web of Science | TS = lumbar disc herniation AND TS = ozone AND TS = steroid  (Identified 34 articles, Date: July 20. 2026. |
| Wanfang | TI＆KY = lumbar disc herniation AND TI＆KY = ozone AND TI＆KY = steroid  (Identified 12 articles, Date: July 20. 2026. |
