# Supplementary material for: Efficacy of ozone with steroid injection therapy in lumbar disc herniation: meta-analysis
Source: Front Med (Lausanne). 2026 Aug 17;13:1891165. doi: 10.3389/fmed.2026.1891165 (PMC13527453; doi:10.3389/fmed.2026.1891165)
Supplement: Supplementary file 2 [file Table_2.docx]

**Supplementary Table 2. Risk-of-bias assessment**

| Study | Pre-intervention | | At intervention | Post-intervention | | | | Overall |
| --- | --- | --- | --- | --- | --- | --- | --- | --- |
|  | Confounding | Selection | Classification | Deviations | Missing | Measurement | Report |  |
| Bayram 2025 | M | M | L | M | M | M | L | M |

M: moderate risk of bias; L: low risk of bias
